# P300 Modulation *via* Transcranial Alternating Current Stimulation in Adult Attention-Deficit/Hyperactivity Disorder: A Crossover Study

**DOI:** 10.3389/fpsyt.2022.928145

**Published:** 2022-07-18

**Authors:** Kyra Kannen, Behrem Aslan, Cindy Boetzel, Christoph S. Herrmann, Silke Lux, Helena Rosen, Benjamin Selaskowski, Annika Wiebe, Alexandra Philipsen, Niclas Braun

**Affiliations:** ^1^Department of Psychiatry and Psychotherapy, University Hospital Bonn, Bonn, Germany; ^2^Experimental Psychology Lab, Department of Psychology, Carl von Ossietzky University, Oldenburg, Germany

**Keywords:** P300, attention deficit/hyperactivity disorder, ADHD, transcranial alternating current stimulation, tACS, therapy

## Abstract

**Objective:**

A repeated finding regarding event-related potentials (ERPs) is that patients with ADHD show a reduced P300 amplitude. This raises the question of whether the attention of ADHD patients can be increased by stabilizing the P300. Assuming that the P300 is generated by event-related oscillations (EROs) in the low frequency range (0–8 Hz), one approach to increase the P300 could be to stimulate the patient’s P300 underlying ERO by means of transcranial alternating current stimulation (tACS). The aim of this follow-up study was to investigate this hypothesized mechanism of action in adult ADHD patients.

**Materials and Methods:**

Undergoing a crossover design, 20 adult ADHD patients (10 female) received an actual stimulation *via* tACS on one day and a sham stimulation on another day. Before and after each intervention, EEG characteristics (P300 amplitudes, low frequency power) and attention performances (d2 attention test, visual oddball task (VOT)) were recorded.

**Results:**

Electrophysiological analyses revealed no evidence for an enhanced P300 amplitude or low frequency power increase after actual stimulation compared to sham stimulation. Instead, a significant effect was found for a stronger N700 amplitude increase after actual stimulation compared to sham stimulation. Consistent with the P300 null results, none of the examined neuropsychological performance measures indicated a tACS-induced improvement in attentional ability.

**Conclusion:**

Contrary to a previous study using tACS to modulate the P300 in adult ADHD patients, the current study yields no evidence that tACS can increase the P300 amplitude in adult ADHD patients and that such P300 enhancement can directly improve neuropsychological parameters of attention.

## Introduction

Attention-deficit/hyperactivity disorder (ADHD) is a common developmental disorder that persists into adulthood, and is associated with core symptoms of inattention, hyperactivity, and impulsivity (1). With an estimated global lifetime prevalence of 2.58% (2), ADHD causes not only severe individual suffering such as difficulties in academic career (3, 4), occupational burdens (5–11) and difficulties in social interactions and relationships (12–19), but also a high burden for society and economy. Considering not only direct diagnostic and treatment costs, but also secondary follow-up costs (e.g., productivity losses due to inability to work or early retirement, justice system costs), the global total annual costs of ADHD are estimated to be at least 831 million [for a systematic review, see (20)]. Therefore, the treatment of ADHD is not only important to reduce individual suffering, but also to avert economic damage.

So far, ADHD is primarily treated by psychostimulants, cognitive behavioral therapy, or a combination of both (21). Although stimulant medication is thereby usually considered as first-choice treatment (22–24), it often leads to undesirable side effects such as sleep disturbances (25), decreased appetite and weight decrease (26) or cardiovascular effects (27). Moreover, in a significant subgroup of ADHD patients, psychostimulants have no, or no sufficient treatment effect (28–30). Also, some patients develop tolerances to psychostimulants (31) and often interrupt or discontinue their medication (32), particularly due to adverse events (33). Consequently, the development of further, effective ADHD therapy approaches with fewer side effects is urgently required.

One explanatory factor for individual differences in response to psychostimulants may be the high pathophysiological heterogeneity within the ADHD population [for a critical discussion, see (34)]. Various combinations of environmental and genetic factors, for instance, lead to diverse neuropsychological impairments and thus to different ADHD symptom profiles (35). Consequently, great research effort is currently being undertaken to identify ADHD biomarkers that are of predictive value for ADHD treatments and could guide practitioners in deciding which treatment options hold most promise in each individual case [for a systematic review, see (36)]. Similarly, there is hope that the discovery of reliable biomarkers helps to develop new treatment approaches that directly target the pathomechanisms revealed by the biomarkers and are not merely symptom-driven.

One such biomarker that might prove useful as a target site in ADHD treatment is the P300 component in electroencephalographic event-related potentials (ERPs) (37). The P300 is a positive voltage deflection around 300 ms after a target stimulus over centro-parietal regions and associated with attentional allocation and stimulus processing (38, 39). Reliable elicitation of the P300 can be achieved, for example, by oddball paradigms, in which subjects are required to respond to infrequent target stimuli and to ignore frequent distractor stimuli (40). Probing such oddball paradigms in ADHD, several studies have found a reduced P300 amplitude (41–48) and prolonged latency (44, 49–53) in adult ADHD patients compared to typically developed individuals. In addition, several research groups report increased P300 amplitudes along with attention improvements after administration of ADHD medication (54–57) or mindfulness-based cognitive behavior therapy (MBT) (58). Hence, the P300 appears to be a reasonable target site for the exploration and development of further therapeutic methods.

If the P300 is abnormally altered in ADHD patients but normalizes after psychostimulant administration or MBT, the question arises whether an attention improvement is also achievable by a direct modulation of the P300, e.g., by applying transcranial alternating current stimulation (tACS). tACS is a non-invasive technique in which the brain is stimulated *via* an alternating current of a beforehand determined frequency. As certain can be considered that tACS can modulate endogenous brain oscillations and, more importantly, cognitive processing [for review, see (59)]. Regarding attentional processing, for instance, an improved accuracy in conjunction search after alpha tACS (i.e., a stimulation frequency around 8 to 12 Hz) (60) and an improved voluntary top-down attention after gamma tACS (i.e., a stimulation frequency >30 Hz) (61) has been reported.

During tACS, the presumed mechanism of action is mainly attributed to the entrainment of intrinsic brain oscillations to the external stimulation signal (59, 62, 63). Entraining oscillations is observed to be most efficient when the frequency of the applied current is close to the intrinsic brain frequency (64). The administered current alters internal neuronal excitability by causing changes in the resting potential (65). Whether neuronal excitability is thereby enhanced or weakened, and consequently increases or decreases the probability of neural firing, is determined either by depolarization or hyperpolarization (66). Taken together, when tACS is applied, the external sinusoidal force and the internal neural firing patterns are synchronized. Moreover, tACS is thought to induce changes in synaptic plasticity (67–69). Whether the synaptic activity between neurons is intensified or attenuated is thereby determined by the timing of the neurons’ input and output activity (pre- and post-synaptic events). TACS can affect this spike probability of neurons and it is believed that these synaptic changes persist after cessation of stimulation, leading to increased power at the chosen stimulation frequency (70–72). This phenomenon is called spike-timing-dependent plasticity [for further details, see e.g., (73)].

Whether tACS can also modulate ERPs is less validated. While the few existing empirical studies on this issue (74–78) yielded mixed results, at least from a theoretical perspective such modulability appears expectable, given that ERPs can be regarded as event-related oscillations (ERO) (79). The P300 component at issue here, for instance, has been closely linked with an ERO in the delta (0–4 Hz) to theta (4–8 Hz) range (80–84). Therefore, at least theoretically, tACS appears to offer a promising therapeutic approach to modulate not only oscillations but also ERPs in ADHD patients.

Despite this high potential tACS may have for the treatment of ADHD, the use of tACS in ADHD has so far little been studied. In fact, consistent with the findings of a recent review of neurostimulation in ADHD (85) that found 30 studies, but none of which applied tACS, our own literature search only yielded one study recently published Dallmer-Zerbe et al. (75) and another study recently published by Farokhzadi et al. (86). In the study by Farokhzadi et al. (86), treatment with 10 Hz alpha tACS was compared to psychostimulant treatment in 62 ADHD children. Over the course of 8 weeks, one group received alpha tACS thrice a week for 10–15 min at pre-frontal electrode sides, while another group received psychostimulant treatment over the same course of time. The reported result is that tACS was more effective than psychostimulant treatment in improving attention and impulsivity, as assessed by the “integrated visual and auditory test.” Although promising, one methodological problem with this result is that it is only based on behavioral, but not on neurophysiological investigations (i.p. an investigation of the EEG alpha spectrum). Therefore, it cannot be ruled out that the group differences found are due to some other mechanisms (e.g., more social devotion during the tACS than psychostimulant intervention) rather than being due to the assumed electrostimulative mechanism of action.

In the study by Dallmer-Zerbe et al. (75), in turn, 18 adult ADHD patients either underwent tACS or placebo stimulation for approximately 20 min. TACS was thereby applied at the participant’s individual ERO, and the presentation of the target stimuli was timed in such a way that the participant’s induced P300 always coincided with the positive voltage peaks of the ongoing tACS. Results showed a significant enhancement of the P300 amplitude in the stimulation group and a tACS-induced decrease in omission errors (75). Also this study had, however, some methodological flaws. In particular, the implemented oddball task turned out to be too easy, so that hardly any errors were committed. Moreover, a between-subjects design was used with only 8 patients per group. Hence, the study might have been underpowered.

Therefore, the aim of the current study was to replicate overall study findings by the previous study by Dallmer-Zerbe et al. (75), and consequently to investigate to what extent tACS can modulate the target P300, the low frequency range, and neuropsychological test performances in adult ADHD patients. To this end, we carried out a crossover study with two separate measurement days in which our 20 adult ADHD patients received a placebo stimulation (sham) in one case and an actual tACS in the other, while conducting an optimized visual oddball task (VOT). Using a mobile EEG system, individual stimulation parameters were determined and individually adjusted on site, using a time-frequency decomposition of the P300. We revised several aspects of the former study by Dallmer-Zerbe et al. (75) like, for example, we used a crossover study design instead of between-subjects design or adjusted the VOT to increase task difficulty (a detailed list comparing both experiments can be found in the Supplementary Table 1).

## Materials and Methods

### Participants

A total of 22 ADHD patients (11 female, *M*_*age*_ = 28.55, *SD* = 8.77, age range: 19–48) volunteered in this study, out of which 20 underwent the entire experiment. All participants were recruited *via* the specialized outpatient clinic for adult ADHD of the Clinic for Psychiatry and Psychotherapy of the University Hospital Bonn. Participants were either personally invited to the study during medical consultations or contacted *via* a study applicant pool in which they had previously registered. A brief telephone screening was then conducted with each study prospect, and if there were no reasons for exclusion, the patient was allowed to participate in the study. Written informed consent was obtained from all participants and they all received an expense allowance of 30 € for their participation. Moreover, the study was approved by the medical ethics committee of the University of Bonn (protocol number: 357–19) and pre-registered at the German Clinical Trials Register (Trial-ID: DRKS00020828).^1^

### Study Design and General Procedure

The study was carried out on three measurement days and as a crossover study with two interventions. The two interventions compared “actual stimulation” and “sham stimulation.” On Day 1, a comprehensive clinical examination was performed, during which the ADHD diagnosis was validated, and the patient’s mental state was evaluated. On Days 2 and 3 in turn, the actual experiment took place, with one of the two conditions being run on each measurement day. While fifty percent of the participants underwent the actual stimulation first on Day 2 and the sham stimulation on Day 3, the remaining fifty percent underwent the sham stimulation first on Day 2 and the actual stimulation on Day 3.

### Eligibility Assessment and Clinical Characterization

All participants were already diagnosed with ADHD or were in the process of diagnosis at our specialized outpatient clinic for adult ADHD. To confirm the ADHD diagnoses and further characterize their individual ADHD symptom profiles, all participants underwent the structured clinical “Interview of Integrated Diagnosis of ADHD in Adulthood” [IDA-R; (87)]. Moreover, to clarify potential comorbidities and exclusion criteria, the German version of the “diagnostic short interview for mental disorders” [Mini-Dips-OA; (88)] was carried out. Likewise, participants filled in four further self-rating questionnaires:

– *Demographic questionnaire*: A lab-internal, self-designed questionnaire that gathered some biographical data (birth, gender, education, family status) relevant for the study.
– *ADHD Self-Report-Scale* [*ADHS-SB*; (89)]: The ADHS-SB is a 22-item questionnaire that surveys key symptoms of ADHD and allows to derive three domain-specific scores (inattention, hyperactivity, impulsivity) and one overall ADHD score.
– *Depression-anxiety-stress-scales* [*DASS-21*; (90)]: A short 21-item questionnaire that assesses indications of depression, anxiety, and stress. For each symptom area, a separate score from 0 (no burden at all) to 21 (maximum burden) may be calculated.
– *WHO quality of life scale questionnaire–short version* [*WHOQOL-Bref*; (91)]: A 26-item questionnaire assessing quality of life in the past 4 weeks in four main domains (physical health, psychological health, social relationships, and environment). To be eligible for the study, participants needed to be right-handed [according to the Edinburgh Handedness Inventory; (92)], to be between 18 and 50 years old, and to have corrected-to-normal or normal vision. In addition, any of the following exclusion criteria had to be absent: Presence of a severe comorbid affective disorder (mild to moderate was included), any psychosis or substance dependence, current use of any psychotropic medication other than ADHD medication, presence of a serious neurological disorder (especially epilepsy), presence of a dermatological disorder of the head, or pregnancy.

### Experimental Procedure

**FIGURE 1:**
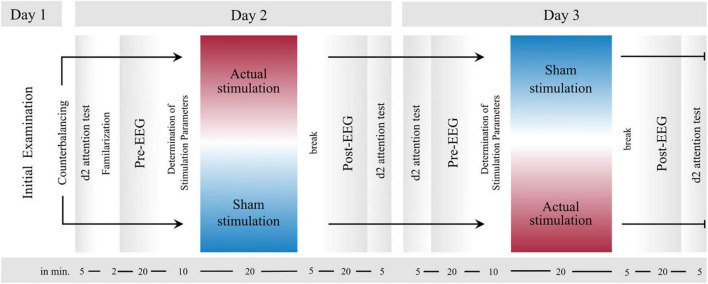
Experimental Design. After an initial examination (Day 1), the two experimental sessions (Day 2 and Day 3) proceeded in the same order, except for a brief familiarization with the visual oddball task (VOT, cf. section “Visual Oddball Task”) in the first experimental session. First, the d2 attention test was performed before all electrodes were attached to the participants’ head. Next, the first block (pre-intervention) started, in which participants accomplished the VOT. Immediately thereafter, there was a short break, during which the tACS parameters were computed from the EEG data of the first block. As soon as these parameters were collected, the second block (intervention) started, during which actual stimulation or sham stimulation was applied. Finally, the third block (post-intervention) started after a short break. During all three blocks EEG was measured. Last, the d2 was conducted again.

Except for the stimulation method applied (actual stimulation vs. sham stimulation) and a short familiarization with the VOT at the first experimental session, the experimental procedure on Day 2 and 3 was identical (cf. Figure 1). Whether participants first received the actual or sham stimulation was counterbalanced across all participants. While participants knew that on one session, they would receive a placebo stimulation and on the other session an actual stimulation, they were kept uninformed about the order of stimulation procedures. On both days of measurement, ADHD medication had to be discontinued 24 h beforehand. For both measurement days, the experiment took place in the Virtual Reality laboratory of the University Hospital of Bonn and the experimental procedure was as follows: First, to record their momentary attention level, participants performed the d2 attention test (d2; cf. section “d2 Attention Test”). Next, the participants were prepared for the actual stimulation or sham stimulation and concomitant EEG measurement. In both experimental sessions, the preparation procedure was thereby identical. After that, the actual experiment started, which consisted of three experimental blocks: a *pre-intervention block*, an *intervention block*, and a *post-intervention block*. The three experimental blocks were each separated by 5- to 10-min breaks (depending on the duration of the online EEG analysis). EEG was recorded throughout blocks and a VOT (cf. section “Visual Oddball Task”) had to be performed in each of the three blocks. The only difference between the three blocks was that during the intervention block, actual stimulation or sham stimulation was applied. To customize the electrical stimulation, the participants’ individual frequency of ERO and P300 peak latency was determined (cf. section “Online Analysis”) in the first short break immediately before the intervention block. As soon as the stimulation parameters were determined, the intervention block with either actual stimulation or sham stimulation started (for details, see section “Synchronization Between Stimulus Presentation and Transcranial Alternating Current Stimulation”). From here on, the experimenter could no longer be blinded to intervention since the stimulator had to be operated manually according to either the sham stimulation or actual stimulation. After the intervention block and a further short break, the last post-intervention block started. Finally, after finishing all three experimental blocks, participants again completed the d2 and filled in a questionnaire assessing adverse effects of tACS (93). In total, the experimental procedure took approximately 2.5 to 3 h, including preparation time for attaching tACS and EEG electrodes.

### d2 Attention Test

As stated, the d2 (94) was applied before and after the three experimental blocks to compare the participant’s individual attention and concentration performances before and after intervention. In accordance with the test manual, the d2 was thereby administered as a paper-pencil test. That is, participants had to cross out target symbols (letter “d” with two strokes) between distracting non-target stimuli (letter “d” with one, three, or four strokes and letter “p” with one, two, three, or four strokes) through 14 consecutive lines of 47 characters each. They were instructed to cross out as many target symbols as possible within a time limit of 20 s per line. Between these 20 s phases, there was no pause, so that the total test time was less than 5 min. To evaluate d2 test performances, the following performance metrics were calculated: the total number of characters processed (as a measure of processing speed), the d2 concentration performance (i.e., the number of correctly identified characters minus all conducted errors), commission errors (i.e., deleted non-target characters), and omission errors (i.e., missed target characters).

### Visual Oddball Task

In all three blocks, the VOT was conducted for about 20 min. Participants sat on a chair 70 cm away from a computer screen on which the oddball task was presented. Stimuli were displayed *via* NBS Presentation (Version 21.0 build 06.06.19, Neurobehavioral Systems Inc., Albany, CA, United States) and logged together with keyboard inputs *via* Lab Streaming Layer (LSL)^2^.

On the center of a gray computer screen, 2° to the left or right tilted gabor stimuli (∼ 4 cm × 4 cm) were iteratively displayed, each with a duration of 500 ms. In total, 400 gabor stimuli were presented, out of which 300 (75%) represented standard stimuli and 100 (25%) target stimuli. Whether the left-tilted or right-tilted gabor stimuli represented the standard stimuli, and thus were presented thrice as often, was counterbalanced across all subjects. That is, in 50% of participants, the left-rotated gabor stimuli represented the frequent standard stimuli throughout measurement days, while in the remaining 50%, they represented the infrequent target stimuli. The ISI between the gabor stimuli was jittered between 1,000 and 2,500 ms. During the intervention block, the target stimulus onset was adjusted so that the peak of the individual mean P300 amplitude coincided with the positive peak of the tACS signal (details below). The participants’ task was to press a key with their left index finger upon each left-rotated stimulus and a key with their right index finger upon each right-rotated stimulus. Thereby, they were requested to execute their keyboard presses as quickly as possible and as accurately as possible and to fixate onto a fixation circle displayed on the computer screen throughout the task. For assessing VOT performances, four main parameters of interest were extracted for each participant: omission error rate (i.e., the percentage of non-target button responses to target stimuli), commission error rate (i.e., the percentage of target button responses to standard stimuli), d-Prime [i.e., a sensitivity measure, calculated by *d*’ = z(Hit Rate)–z(False alarm rate)] and mean reaction time (RT, mean reaction time of the correct target responses). While the omission error rate is considered as a measure of inattention, the commission error rate is thought to reflect impulsivity (95).

### Electrical Brain Stimulation and Electrode Montage

Electrical stimulation was only administered during the intervention block using a battery-operated stimulator system (DC-stimulator plus, Neuroconn, Ilmenau, Germany). In total, four 7 cm × 3.5 cm rubber electrodes were placed on the participant’s head, whereby two of them were placed above C1/C2 and the other two above C5/C6 (for orientation of the electrodes, see Supplementary Figure 1). The electrode montage was selected based on a simulated finite-element model of current flow. More specifically, using the ROAST Toolbox (96) and the MNI standard brain as template, different electrode montages were simulated in respect to their predicted intracranial electrical field in parietal and temporal regions (i.e., the region, where the P300 is most prominent) (97). The selected electrode montage thereby offered the best compromise between the requirement to generate a high intracranial current flow in the target region and the requirement to avoid blocking any EEG electrodes relevant for the EEG analyses. A graphical illustration of the conducted electrode montage simulation may be found in the Supplementary Figure 1. The four tACS electrodes were applied using conductive paste (Ten20 conductive paste, Weaver and Co, Aurora, CO, United States), and for all participants, impedances were kept below 10 kΩ.

For the actual stimulation condition, tACS was applied for about 20 min, with an intensity of 1 mA (peak-to-peak). The previously conducted electric field simulation with an injected current of 1 mA peak-to-peak per electrode pair yielded to an electric field strength of ∼ 0.1 V/m (Supplementary Figure 1). Previous studies showed [c.f. e.g., (98)] that similar electric field strengths in the target area produced aftereffects. The stimulation frequency was individually adjusted for each participant and reflected the participants’ individual frequency peak between 1 and 8 Hz during target trials (details below). To minimize discomfort, the stimulation was faded in and out for about 10 s. For the sham stimulation, in turn, tACS was again faded in for about 10 s, but then only lasted for another 10 s, before it was again faded out for 10 s. Hence, in total, the “tACS” during the sham stimulation conditions only lasted for 30 s including fade-in and fade-out phases and served the purpose of realistically mimicking the phenomenological experience of actual stimulation. This procedure is one of the commonly used placebo stimulation techniques [e.g., (99)]. To identify potential differences in the perception of both conditions, at the end of each session participants were asked whether they received actual or sham stimulation, and whether they perceived any tACS side effects (93).

### Synchronization Between Stimulus Presentation and Transcranial Alternating Current Stimulation

**FIGURE 2:**
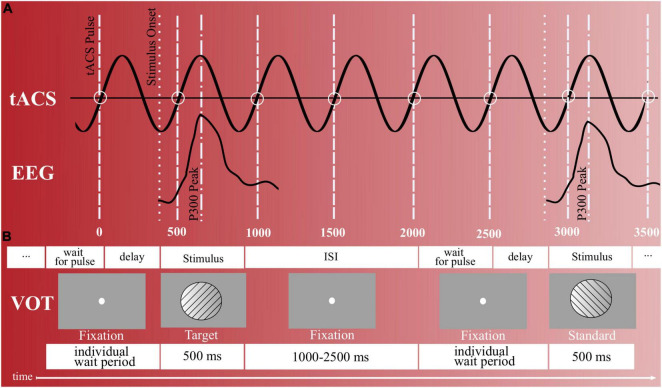
Stimulus presentation and timing. **(A)** Synchronization between target trials and tACS peaks during the intervention block (actual stimulation). To coincide the participant’s elicited P300s with the tACS’s positive voltage peaks, NBS presentation waited for a pulse of the stimulator (wait for pulse). In addition, an individual delay existed that delayed until the tACS waveform was at the specific position so that its next positive peak would coincide with the next P300 peak triggered by a target stimulus. **(B)** Visual oddball task with right- and left-tilted gabor stimuli. Upon each left-rotated stimulus, participants were to respond with a left-hand button press and upon each right-rotated stimulus with a right-hand button press. In total, 400 gabor stimuli were presented, out of which 25% represented target stimuli and 75% standard stimuli. ISI = inter stimulus interval.

To always coincide each participant’s individual target P300 during the intervention block with a positive voltage peak of the running tACS, a similar synchronization approach was used as in the previous study (75) (cf. Figure 2A). As the internal oscillation is believed to synchronize with the external tACS force and to thereby enhance its power, in-phase tACS (internal oscillation frequency matches with external force) is reported to synchronize EROs, while anti-phase tACS (internal oscillation frequency does not match with external force) is reported to desynchronize EROs [for a discussion, see (100)]. That is, the presentation of the next stimulus was paused by a waiting period until a pulse of the stimulator signaled that the tACS waveform was at a certain position that its next positive peak would coincide with the next P300 peak triggered by the stimulus (cf. Figure 2). During this wait period, a fixation point was shown. Technically, this was realized by transmitting the pulse from the stimulator to NBS Presentation at the beginning of each new sinusoidal wave (i.e., upon each zero crossing in the sinusoidal’s ascending flank). Based on this, it was possible to define when the next positive tACS peak would occur and thereby adapt the delay for showing the stimuli (cf. Figure 2B). This calculation thereby considered both, the fixed P300 latency and individual stimulation frequency, which were already determined during the VOT pre-intervention block (cf. see section “Online Analysis”).

### Electroencephalography Recording and Analysis

Electroencephalography (EEG) was acquired *via* a wireless EEG system (Smarting^®^, mBrainTrain^®^, Belgrade, Serbia) from 22 Ag/AgCl sintered ring electrodes (Fp1, Fp2, AFz, F3, Fz, F4, T7, C3, Cz, C4, T8, CPz, P7, P3, Pz, P4, P8, POz, O1, O2, M1, M2 according to the international 10/20 system). FPz served as ground (DRL) and FCz as reference electrode (CMS). The amplifier was attached to the EEG cap (Easycap, Herrsching, Germany) and communicated wirelessly with the recording computer *via* Bluetooth. Keeping all impedances below 15 kΩ, the EEG was digitized at 500 Hz (one data set was unintentionally recorded at 250 Hz) and with a 24-bit step-size resolution *via* (LSL). The marker stream originating from NBS Presentation was thereby also acquired *via* LSL, such that the EEG recording files entailed all event information of the conducted VOTs. Data analysis was performed using Matlab 2021b (The MathWorks Inc., Natick, MA, United States) and eeglab 2021.0 (101).

#### Online Analysis

For the on-site EEG analysis during the experiment, the participant’s EEG data from the pre-intervention block was filtered with a 40 Hz low-pass filter and a 0.1 Hz high pass filter, and then detrended. Next, before the computation of an independent-component-analysis (ICA) the continuous EEG data was epoched into 2 s time windows. After that, a fast ICA was computed using pop_runica (ica type “fastica”) on the epoched EEG data and its components were visually inspected. ICA components reflecting obvious artifacts (e.g., horizontal or vertical eye movements, heartbeats, muscle activity or electrode artifacts) were identified, backprojected to the filtered continuous EEG data, and then rejected. Next, for the calculation of the P300 peak latency, the ICA-corrected continuous EEG data was first epoched from −2 to + 5 s relative to each target stimulus, and then baseline-corrected beginning from −2 s until target onset. Remaining non-stereotypic artifacts were removed by built-in EEGLAB functions (kurtosis thresholding and joint probability test with ± 3-SD single-channel and global-channel thresholds). Then, the participant’s P300 latency was derived by averaging all epochs for electrode Pz and identifying the maximum P300 amplitude peak between 250 and 450 ms after target stimulus onset.

The participant’s most dominant event-related oscillation during the P300 time window, in turn, was determined by a frequency analysis. First, using Matlab’s pspectrum function, the power spectrum at electrode Pz was calculated for each epoch and then all derived power spectra were averaged to obtain one mean power spectrum. The obtained frequency resolution was 0.1 Hz and the obtained time resolution 0.124 ms. Next, the highest frequency power within the time frame of ± 200 ms around the previously determined P300 latency and within the frequency range of 1 and 8 Hz was determined and used as the individual stimulation frequency.

#### Pre-processing and Data Cleaning

For the EEG offline analyses, the EEG datasets from the pre-intervention and post-intervention block were first merged, down-sampled to 250 Hz, temporally filtered between 0.5 and 40 Hz, and detrended.

In three datasets, noisy EEG channels (max. 3) were identified and replaced *via* spherical interpolation using the pop_interp function. For one dataset, a 1.1 s long highly artifactual data segment was removed. Next, for the computation of an ICA, the continuous EEG data was segmented in 2 s time windows and non-stereotypic artifacts were removed using built-in EEGLAB functions (joint probability test, ± 2-SD single-channel and global-channel thresholds). After that, an ICA (“extended” version) was computed and components reflecting horizontal or vertical eye movements, heartbeat, muscle activity or electrode artifacts, were visually identified, backprojected to the continuous EEG data and then rejected. Hence, at the end of this cleaning process, continuous EEG data sets were obtained that were already filtered between 0.5 and 40 Hz and cleaned from stereotypic artifacts by means of the conducted ICA.

#### Event-Related Potentials Analyses

Event-related potentials analyses focused on differences in the target P300 between interventions (actual stimulation vs. sham stimulation) and blocks (pre-intervention vs. post-intervention block). To this end, the merged and ICA-corrected continuous EEG datasets for each intervention were first rereferenced to the common average, low-pass filtered below 6 Hz (to exclude alpha activity), epoched from −0.5 to 1.5 s relative to each target stimuli, and then cut into two separate subsets: One subset containing the epochs of the pre-intervention block before actual stimulation or sham stimulation, another subset containing the epochs of the post-intervention block after actual stimulation or sham stimulation. Next, the same following pre-processing and analysis steps were performed on each subset: First, a baseline correction was applied on each epoch by subtracting the mean voltage of the −0.5 s epoch prior to stimulus onset from all data points. Second, within each epoch, channels that exceeded a differential average amplitude of 150 μV were marked for rejection. Channels that were marked as bad on more than 15% of all epochs were excluded. Epochs having more than 10 bad channels were excluded, while epochs with less than 10 bad channels were included. The bad-channel data was replaced with spherical interpolation of the neighboring channel values [TBT, (102)]. Third, the ERP of the respecting condition was calculated by taking the average across epochs. Finally, for the statistical analyses, for each dataset, the mean P300 amplitude was calculated for electrode Pz within the time range from + 200 to + 550 ms. In addition, the maximum P300 peak between 250 and 550 ms was extracted for each dataset. The same processing procedure was implemented for inspecting the standard P300.

#### Frequency Analysis

The frequency analyses focused on spectral differences in the delta to theta range between interventions and blocks. To this end, the ICA-corrected continuous EEG datasets for each condition were again rereferenced to the common average, epoched from −0.5 to + 1.6 s relative to each target stimulus, and then cut into two subsets for pre- and post-block measurements. Next, the identical following pre-processing and analysis steps were performed on each subset: First, a baseline correction was applied from −0.5 to 0 s, before the same non-stereotypic artifact removal was implemented as described for the P300 analysis. Next, a continuous wavelet transformation (CWT) was conducted on each retained epoch for channel Pz. The frequency range obtained reached from 0.25 to 6 Hz in 47 steps on a log scale and the time resolution amounted to 0.004 ms. After that, the derived power spectra were logarithmized and a mean power spectrum was derived by averaging across all derived power spectra. Finally, for the statistical analyses, the mean delta and theta (0.5–5.5 Hz) power of the respecting subset (condition) was derived by taking the average power across all frequency bins falling into the respecting frequency range and time range between 250 and 550 ms.

### Statistical Analyses

Two participants had to be excluded after the first diagnostic appointment, one because of meeting the exclusion criteria and another one due to health problems. Additionally, out of the 20 participants who completed the entire experiment, one participant had to be excluded from the analyses due to incorrect task execution. Hence, 19 participants remained for further analyses from which the following outcome variables were extracted: Omission error rate, commission error rate, mean RT and reaction time variabilities (RTV) for the VOT analyses; processing speed, omission errors, commission errors and concentration performance for the d2; target P300 mean amplitudes for the ERP analyses; and low frequency power values for the wavelet analysis.

For each main dependent variable, a two-way repeated measures ANOVA with the two within-factors “Block” (pre-intervention vs. post-intervention) and “Intervention” (actual stimulation vs. sham stimulation) was conducted. For specifying ANOVA effect sizes, partial eta squared (η_*p*_^2^) was used, where η_*p*_^2^ = 0.01 indicates a small effect, η_*p*_^2^ = 0.06 a medium effect, and η_*p*_^2^ = 0.14 a large effect (103). For indicating effect sizes of *t*-tests, on the other hand, Cohen’s d was used, where *d* = 0.20 indicates a small effect, *d* = 0.50 a medium effect, and *d* = 0.80 a large effect (103). The α-level was set to 0.05.

In addition, to identify potential associations between the different outcome parameters, exploratory Pearson correlation analyses between each possible variable pair were conducted on the absolute change (difference from pre-to-post) across both intervention types. Correlation analyses were tested for significance and Bonferroni-Holm correction was applied to correct for multiple comparisons. All statistical analyses were carried out using Matlab (The MathWorks Inc., Natick, MA, United States, Version 2021b).

## Results

### Sample Characteristics

Sociodemographic and clinical characteristics of the finally analyzed sample are reported in Table 1. 57.89% of participants were diagnosed with the combined ADHD type, 5.26% with the predominantly hyperactive-impulsive subtype and 36.84% with the predominantly inattentive ADHD subtype. The most common current comorbidities found were anxiety disorders (36.84%) and affective disorders (21.05%). According to the DASS-21 (90), participants revealed, on average, only mild scores for depression (*M* = 10.26; *SD* = 3.48), anxiety (*M* = 9.11; *SD* = 2.45) and stress (*M* = 12.53; *SD* = 5.65). On average, participants were 27.95 years (*SD* = 8.57) and most participants had a higher education entrance qualification (78,95%). After each experimental session, participants were asked to judge if they were actually stimulated with tACS or if they received the sham stimulation. 47,37% of the sample correctly judged that they received actual stimulation at the actual stimulation session, while 52,63% thought they were actually stimulated at the sham stimulation session. Since it was a 50% chance to correctly identify the actual stimulation, participants seemed to be blinded.

**TABLE 1:** Sociodemographic and clinical sample characteristics.

Total sample (*n*):		19*	
Female [*n* (*%*)]:		10 (52.63)	
Age [*M* (*SD*)]:		27.95 (8.57)	

Interview data:			

**IDA-R**			Maximum reachable scores:
ADHD presentations [*n* (%)]			
Combined type		11 (57.89)	
Predominantly hyperactive-impulsive type		1 (5.26)	
Predominantly inattentive type		7 (36.84)	
ADHD scores [*M* (*SD*)]			
Total		36.42 (9.14)	54
Inattention		21.58 (3.04)	27
Hyperactivity		7.32 (5.08)	15
Impulsivity		7.53 (3.99)	12
**Mini-DIPS**			
*n* (%)	Current diagnosis	Previous diagnosis	
Affective disorder	4 (21.05)	5 (26.32)	
Anxiety disorder	7 (36.84)	1 (5.26)	
Post-traumatic stress disorder	0	2 (10.53)	
Obsessive-compulsive disorder	3 (15.79)	0	
Sleep disorder	3 (15.79)	1 (5.26)	
Impulsivity Screening	1 (5.26)	4 (21.05)	
Questionnaire data: *M* (*SD*)			
**ADHS-SB**			Maximum reachable scores:
Total		23.53 (11.78)	54
Inattention		12.95 (5.52)	27
Hyperactivity		5.79 (4.95)	15
Impulsivity		4.79 (3.44)	12
**WHOQOL**			Maximum reachable scores:
Total		70.97 (10.46)	100
Physical health		73.12 (10.67)	100
Psychological health		63.16 (15.67)	100
Social relationships		69.30 (16.45)	100
Environment		78.29 (12.61)	100
**DASS-21**			Maximum reachable scores:
Total		10.63 (3.41)	21
Depression		10.26 (3.48)	21
Anxiety		9.11 (2.45)	21
Stress		12.53 (5.65)	21

ADHS-SB, ADHD self-assessment scale; DASS, depression-anxiety-stress-scales; IDA-R, integrated diagnosis of ADHD in adulthood; Mini-DIPS, diagnostic short interview for mental disorders; WHOQOL, world health Organization quality of life questionnaire. *Out of 20 participants who completed the entire experiment, one participant had to be excluded from the analyses due to incorrect task execution. Hence, 19 participants remained for analyses.

### Visual Oddball Task

**FIGURE 3:**
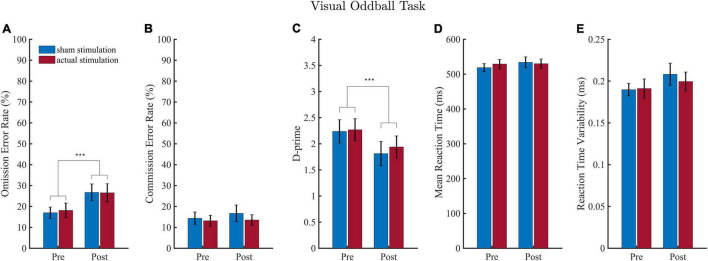
Results of the visual oddball task (VOT). Error rates are depicted in **(A,B)** and the sensitivity measure D-prime in **(C)**. Results of the mean reaction time (RT) and reaction time variability (RTV) are illustrated in **(D,E)**. Values depict means and SEMs for the sham stimulation (blue bars) and actual stimulation (red bars) before and after actual or sham stimulation. ***p < 0.001.

Results of the VOT analyses are shown in Figure 3. Regarding omission error rate (Figure 3A), the ANOVA revealed a significant main effect of “Block” [*F*_(1, 18)_ = 20.13, *p* < 0.001, η_*p*_^2^ = 0.53], but no main effect of “Intervention” [*F*_(1,18)_ = 0.08, *p* = 0.781, η_*p*_^2^ = 0.00] and no interaction effect [*F*_(1,18)_ = 0.16, *p* = 0.693, η_*p*_^2^ = 0.01]. The block effect consisted of more omission errors being committed during the post-intervention (*M* = 26.63; *SD* = 17.49) than pre-intervention (*M* = 17.55; *SD* = 13.01) block.

Regarding d-Prime (Figure 3C), the ANOVA revealed a significant main effect of “Block” [*F*_(1, 18)_ = 17.85, *p* < 0.001, η_*p*_^2^ = 0.50], but no main effect of “Intervention” [*F*_(1,18)_ = 0.47, *p* = 0.501, η_*p*_^2^ = 0.03] and no interaction effect [*F*_(1,18)_ = 0.32, *p* = 0.576, η_*p*_^2^ = 0.02]. The “Block” effect consisted of a smaller d-Prime sensitivity score during the post-intervention (*M* = 1.88; *SD* = 0.94) than pre-intervention (*M* = 2.25; *SD* = 0.87) block.

For commission error rate (Figure 3B), RT (Figure 3C) and reaction time variability (Figure 3D), the ANOVA yielded neither a main effect of “Block” or “Intervention,” nor an interaction effect (detailed ANOVA tables are shown in the Supplementary Table 2).

### d2 Task

**FIGURE 4:**
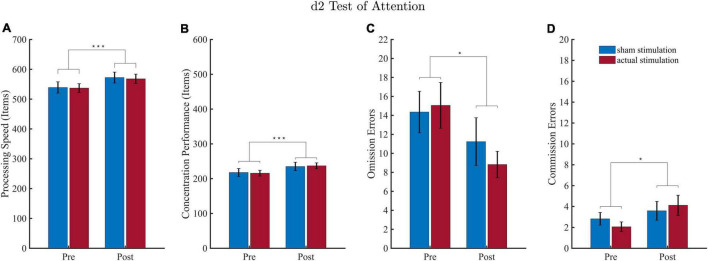
Results of the d2 attention test (d2). Values depict means and SEMs for the sham stimulation (blue bars) and actual stimulation (red bars) before and after intervention. **(A)** Processing speed depicts the total number of characters processed on average for each condition. **(B)** Concentration performance depicts the number of correctly identified characters minus all conducted errors averaged for each condition. **(C**,**D)** Number of omission and commission errors averaged for each condition. *p < 0.05, ***p < 0.001.

Overall performances of the d2 task are depicted in Figure 4. Two datasets had to be excluded due to complications in the execution of the task. For processing speed and concentration performance, there were 2 outliers (>3 SD), and for errors of omission and commission, there was 1 outlier (>3 SD), so that a total of only 16 and 17 datasets, respectively, were included in the respective statistical analyses.

For all d2 performance parameter, the ANOVA revealed a significant block effect. Regarding processing speed (Figure 4A), the effect of “Block” revealed higher processing speed during the post-intervention block (*M* = 570.19; *SD* = 60.48) as compared to the pre-intervention block (*M* = 538.06; *SD* = 59.66). For concentration performances (Figure 4B) the “Block” effect consisted of a higher concentration performance during the post-intervention (*M* = 235.97; *SD* = 36.44) than pre-intervention (*M* = 216.75; *SD* = 34.61) block. For omission errors (Figure 4C) results revealed that less target stimuli were missed during the post-intervention (*M* = 10.03; *SD* = 6.91) than pre-intervention (*M* = 14.71; *SD* = 8.31) block. Results for commission errors (Figure 4D) yielded that more stimuli were wrongly identified as a target during the post-intervention (*M* = 3.85; *SD* = 3.08) than pre-intervention (*M* = 2.44; *SD* = 1.69) block.

There was neither a significant effect for “Intervention,” nor an interaction effect for all four d2 performance parameter (detailed ANOVA tables are shown in the Supplementary Table 3).

### Analyses of Event-Related Potentials

#### Planned Analysis of the Event-Related Potential P300

**FIGURE 5:**
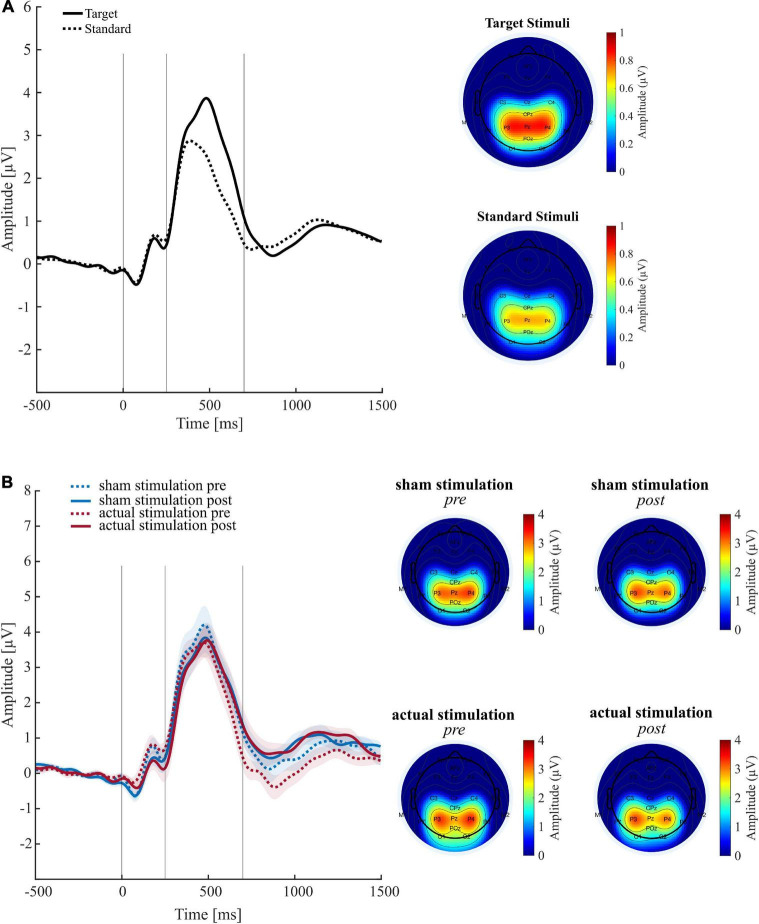
Results of the event related potential (ERP) analyses. **(A)** Grand average ERP waveforms (left panel) and associated topographies (right panel) across all conditions for target and standard stimuli. **(B)** Target P300 ERPs (left panel) and associated topographies (right panel) for each main experimental condition. Shaded curves reflect the standard error of the mean. Time windows for statistical analysis are depicted in the entire 250–550 ms time window.

The topographies and waveforms of the examined ERPs are depicted in Figure 5. Consistent with the literature, extracted ERPs showed the typical waveform and topography of a P300 during an oddball task [for review see e.g., Polich (38)], with a maximum peak at around 250 to 550 ms over centro-parietal electrodes. Moreover, also in agreement with the literature (104, 105), the P300 mean amplitude across conditions turned out to be significantly [*t*(18) = −4.25, *p* ≤ 0.001]) higher for target ERPs than standard ERPs (cf. Figure 5A).

Regarding experimental conditions, the ANOVA on target P300 mean amplitudes revealed a trend for the main effect “Block” [*F*(_1,18)_ = 3.40, *p* = 0.082, η_*p*_^2^ = 0.16] but neither an effect of “Condition” [*F*_(1,18)_ = 0.27, *p* = 0.609, η_*p*_^2^ = 0.01], nor an interaction [*F*_(1,18)_ = 0.03, *p* = 0.870, η_*p*_^2^ = 0.00]. The trend for “Block” consisted of an amplitude decrease during the post-intervention (*M* = 2.72; *SD* = 1.30) compared to the pre-intervention (*M* = 3.00; *SD* = 1.48) block. Individual mean amplitude plots are included in the Supplementary Figure 2. The ANOVA for maximum P300 peak amplitude revealed no significant effects (cf. Supplementary Table 4).

#### Exploratory Analysis of a Late Event-Related Potential

On visual inspection of the ERP waveforms, there appears to be a difference in a late negative ERP component that peaks around 800 ms after target onset (cf. Figure 5B). Therefore, to examine whether this difference is not merely descriptive, we performed an exploratory ERP analysis using the same analysis procedure and the same preprocessed datasets than before, but with a time window of interest slightly shifted backward (700 to 1000 ms). The ANOVA on this late ERP mean amplitudes revealed no main effect of “Intervention” [*F*_(18,1)_ = 0.24, *p* = 0.240, η_*p*_^2^ = 0.08], but a trend for “Block” [*F*_(1,18)_ = 4.03, *p* = 0.060, η_*p*_^2^ = 0.18] that consisted of higher ERP mean amplitudes during the post-intervention (*M* = 0.69; *SD* = 1.29) than pre-intervention (*M* = 0.16; *SD* = 1.48) block. Moreover, the ANOVA revealed a significant interaction [*F*_(1,18)_ = 6.56, *p* = 0.020, η_*p*_^2^ = 0.27]. Following up this effect, paired *t*-tests revealed that the late ERP mean amplitudes significantly increased from pre-intervention (*M* = −0.09; *SD* = 1.14) to post-intervention (*M* = 0.71; *SD* = 1.31) under actual stimulation [*t*(18) = −2.70, *p* = 0.015], but not under sham stimulation [*t*(18) = −0.98, *p* = 0.339].

### Frequency Analyses

**FIGURE 6:**
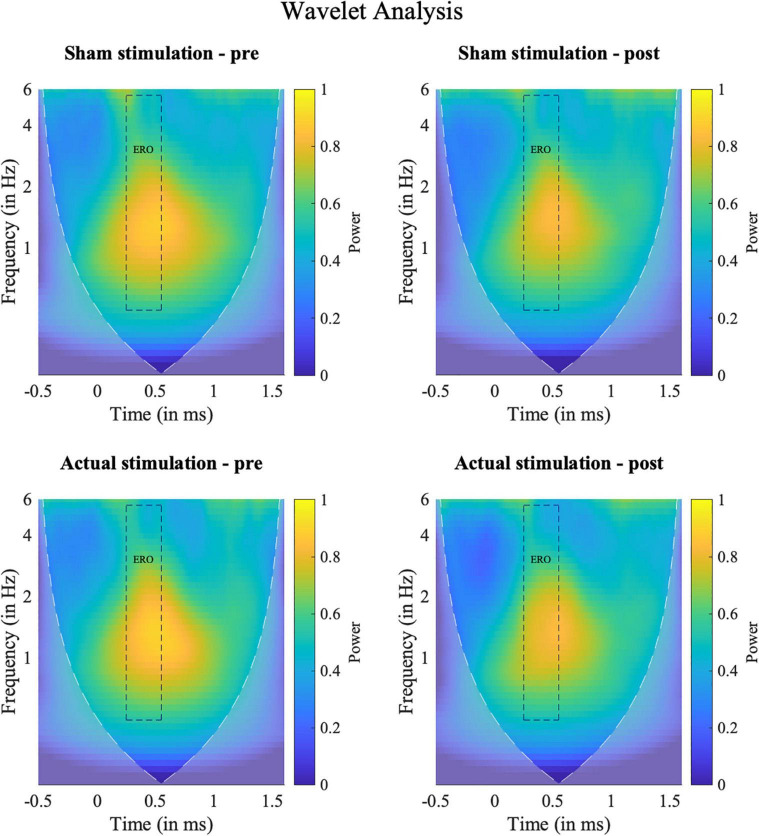
Results of the frequency analyses. Time-Frequency spectra of the wavelet analyses for target hits for the ERO between 0 and 6 Hz at electrode Pz (Grand average). Squares indicate the temporal (0.25–0.5 s) and spectral (0.5–5.5 Hz) region of interest used for the statistical evaluation. Shaded areas outline the area where the calculated wavelet power might be distorted due to edge artifacts.

Time-frequency power spectra of the wavelet analyses are depicted in Figure 6. In line with previous research (74, 75), our wavelet analysis revealed strongest activity in the P300 time window for the ERO in the delta to theta (0–8 Hz) frequency spectrum. The ANOVA on the ERO power values revealed a significant main effect of “Block” [*F*_(1,18)_ = 8.26, *p* = 0.010, η_*p*_^2^ = 0.31], but no main effect of “Intervention” [*F*_(1,18)_ = 0.01, *p* = 0.934, η_*p*_^2^ = 0.00] and no significant interaction [*F*_(1,18)_ = 0.21, *p* = 0.653, η_*p*_^2^ = 0.01]. The “Block” effect consisted of less activity in the ERO band during the post-intervention (*M* = 0.58; *SD* = 0.30) than pre-intervention (*M* = 0.63; *SD* = 0.31) block. Topography plots are shown in the Supplementary Figure 3.

### Explorative Correlation Analyses

Results of the correlation analysis are shown in Table 2. There was a significant positive correlation between the late ERP mean amplitude and VOT RT [*r*(18) = 0.70, Bonferroni-Holm adjusted *p* = 0.045] as well as between the VOT omission error rate and the d prime scores [*r*(18) = −0.89, Bonferroni-Holm adjusted *p* < 0.001]). In addition, there was a significant positive correlation between maximum and mean P300 amplitude [*r*(18) = 0.70, Bonferroni-Holm adjusted *p* < 0.05]. All remaining correlations did not remain significant after Bonferroni-Holm adjustment.

**TABLE 2:** Results of correlations analyses.

	P300 mean amplitude	P300 maximum amplitude	Late ERP mean amplitude	Low frequency Power	VOT omission error rate	VOT commission error rate	VOT dprime	VOT RT	VOT RTV	d2 omission errors	d2 commission errors
Pre-to-Post											
P300 mean amplitude		0.70*	0.13	0.14	0.19	–0.28	–0.15	–0.04	–0.17	0.07	–0.21
P300 max. amplitude			0.36	0.39	0.14	–0.20	–0.14	–0.06	–0.37	0.07	–0.13
Late ERP mean amplitude				0.23	0.20	–0.14	–0.18	0.69*	0.14	0.31	0.01
Low frequency Power					0.17	0.25	–0.37	0.09	0.14	0.23	0.02
VOT omission error rate						0.35	−0.89***	0.33	0.29	–0.12	0.23
VOT comission error rate							–0.56	0.02	0.36	–0.13	–0.23
VOT dprime								–0.34	–0.39	0.12	0.07
VOT RT									0.63	0.07	–0.02
VOT RTV										0.06	–0.24
d2 omission errors											–0.12
d2 commission errors											

Pearson correlations (r) between the absolute change (i.e., the difference from pre to post intervention) for all main behavioral and neurophysiological measures across intervention conditions. Correlations including d2 outcome parameter are calculated with 16, while all others with 19 datasets. *p < 0.05, ***p < 0.001. Bonferroni-Holm correction was applied to correct for multiple comparisons. RT, reaction time; RTV, reaction time variability; VOT, visual oddball task.

## Discussion

In this study, we aimed to increase the P300 amplitude in ADHD patients *via* tACS and to demonstrate an attentional improvement induced by this P300 elevation. Specifically, our hypotheses were (1) that by applying tACS at the participant’s individual ERO, it would be possible to enhance the P300 amplitude in ADHD patients, and (2), that this induced P300 elevation would lead to immediate improvements in neuropsychological attention measures. To test our hypotheses, we subjected our ADHD patients to both, an actual stimulation, and a sham stimulation, and evaluated their EEG characteristics (P300 amplitudes, low frequency power) and attention performances (d2 attention test, VOT) before and after the two interventions.

### No Evidence for a Stimulation-Induced P300 Increase

Contrary to our expectations, we were not able to demonstrate a stronger increase in P300 amplitude under actual stimulation than sham stimulation. Instead, we only found some indication for a tACS-induced amplitude increase in a late ERP component (discussion below). Hence, limited to our analyses and in contrast to the previous study with ADHD patients (75), but in line with another study conducted in healthy participants (74), we currently cannot provide evidence that our methodological approach of aligning the participant’s generated P300 peaks with positive deflections of the tACS signal is able to amplify the P300.

Why we did not succeed in increasing the participants’ P300 through our tACS application cannot be conclusively determined, but some possible reasons can be suggested. First, it should be noted that the effect of tACS may vary due to individual differences in the neuroanatomy, which result in varying electric fields inside the brain (98). Therefore, one explanation might be that despite our careful simulation attempts to find the right electrode montage, we failed to stimulate the correct target region by assuming an inaccurate P300 source location. In the future, it should therefore be considered whether individualized electrode assemblies can be employed, with the help of which individual neuroanatomical peculiarities can be better accounted for.

Likewise, inter- and intraindividual variability in brain activity may have influenced the success of brain stimulation, for example, by an unfavorable brain state during stimulation (106). If this has been the case, a closed loop system that measures brain activity during stimulation *via* EEG and adjusts the applied stimulation accordingly, could potentially provide mitigation here. However, research studies targeting closed loop systems aiming to adapt fluctuating stimulation parameters to momentary brain activity are currently rare and require further investigation (107–110).

Moreover, we find that not only the participant’s P300, but also their event-related low frequency power (0–6 Hz) remained unaffected by our two stimulation interventions. Hence, the reason for failing to increase the P300 could be that the participant’s ERO, which is assumed to be causative of the P300 (80, 81, 83, 84), could not sufficiently be increased. Thus, the question arises why the participant’s ERO has not been changed by tACS. One finding to consider here is that brain oscillations only seem to be increasable by tACS if their power is rather low before stimulation (111, 112). Hence, one possible reason might be that the EROs of our adult ADHD sample were already elevated before the tACS intervention, and therefore could not be further increased. This would be in line with some evidence for an elevated delta and theta power in adult ADHD (113–118), although other studies did not find this effect (119–121). If an elevated delta to theta power in ADHD patients would explain our null finding, the question, however, arises why this effect did not also show up in the previous ADHD study by Dallmer-Zerbe et al. (75) and why the low-frequency power even decreased from pre- to post.

Another reason why we might have failed to enhance the participant’s ERO might be some mismatch between the externally applied tACS frequency and actual ERO. Time constraints during experimental sessions with patients demand a quick EEG data analysis, which may have prevented us from being sufficiently accurate in identifying the participant’s exact ERO. If the external stimulation frequency matches the endogenous frequency, already low stimulation intensities lead to entrainment. However, the larger the variance between internal and external frequency is, the stronger the force of tACS must be to entrain these oscillation (122).

Finally, evaluations of an experiment by Wischnewski et al. (76, 123) indicate that frontal theta tACS (and perhaps this effect also applies to our tACS electrode montage) may induce a P300 drop at least in healthy participants. That is, contrary to their intention of enhancing the participant’s P300 by theta tACS, the participant’s P300 decreased by this intervention. Surprisingly, however, this P300 decrease (76) does not seem to have been caused by modulating the participant’s internal theta power, since it was not affected by the application of tACS (123). One possible implication of this is that there is another indirect mechanism by which an externally applied theta tACS may reduce the P300 amplitude, and perhaps a similar mechanism may potentially also have occurred in our experiment, but further research is required to explore underlying mechanisms.

### Preliminary Evidence for a Stimulation-Induced Late Component Increase

While we found no evidence for a tACS-induced P300 increase, we interestingly found a significant (*p* = 0.020) interaction effect for a late negative ERP component (700–1,000 ms), in that this ERP component was significantly increased after actual stimulation [*t*(18) = –2.70, *p* = 0.015], but not after sham stimulation [*t*(18) = −0.98, *p* = 0.339]. Hence, at least on this ERP component, tACS seems to have had some effect. While we do not yet have a sound neurophysiological explanation on how tACS affected this ERP component, this possible effect clearly warrants further investigation for several reasons. First, previous studies found a relationship between the amplitude of the late negative ERP component N700 and the amount of attention allocated to stimuli (124–126). And second, there is evidence that the N700 amplitude is correlated with a dopamine transporter allele (127) which is considered as a risk factor for ADHD. Consequently, a targeted modulation of this component *via* tACS could also be interesting for the treatment of ADHD.

### No Indication for a Stimulation-Induced Improvement of Attention

In line with the P300 null findings were also the neuropsychological outcomes in our study. For both, the VOT and d2 attention task, none of the assessed performance measures indicated any “Block” × “Intervention” interaction. Altogether, these results suggest that the application of tACS had little to no influence on the measured neuropsychological performance of our participants. This is, however, not surprising, given that the anticipated P300 amplification was already inefficient.

### Successful Optimization of Our Visual Oddball Task

To enhance omission and commission errors, we changed the VOT used in the previous study (75). In particular, we changed the used stimuli, reduced the time period of stimulus presentation and, in addition, the response behavior. Our results suggest that this adaptation of the VOT has been successful in elevating the level of difficulty. In contrast to the previous study with almost no commission errors and a low omission error rate, we now encountered higher omission error rates (*M*_*pre*_ = 17.55%, *SD*_*pre*_ = 13.01% and *M*_*post*_ = 26.63%, *SD*_*post*_ = 17.49%) and commission rates (*M*_*pre*_ = 13.76%, *SD*_*pre*_ = 9.55% and *M*_*post*_ = 15.11%, *SD*_*post*_ = 11.64%), while still observing a plausible P300 ERP (40). For future follow-up studies on the same topic, we therefore propose to use our improved VOT variant instead of our original one.

### Marginal Associations Between Main Experimental Parameters

Most of the major correlation parameters were non-significant. However, there was one significant positive correlation between late ERP mean amplitude and VOT RT [*r*(18) = 0.70, Bonferroni-Holm adjusted *p* = 0.045]. While preliminary, this finding might suggest that the amplitude change of the late ERP component could be influenced by the participant’s RT during the VOT. Therefore, the modulation of this late ERP component could be a future target site to be investigated to influence responsiveness in ADHD individuals.

### Limitations and Future Directions

One limitation of our study is that the experimental design is rather time critical and grounds on the presupposition that the participant’s P300 latency remains stable across trials. If this requirement is violated too strongly, there is a risk that the tACS peaks do not sufficiently coincide with the P300 peaks, and thus the P300 cannot sufficiently be elevated. For the future, this problem could perhaps be attenuated by using an oddball task that induces a particularly low P300 latency variability, choosing a less time-critical target site instead of the P300 (e.g., an oscillation instead of an ERP component), or by implementing a closed loop system that may recognize P300 latency changes over time and may adapt the stimulation frequency accordingly.

In comparison to the study of Dallmer-Zerbe et al. (75), we changed various aspects in our present study. For example, we chose another study design (crossover design instead of between design), we used other electrodes for the application of tACS (rubber electrodes instead of EEG ring electrodes) and programmed a different visual oddball task with different stimuli and reaction patterns (for further details and differences cf. Supplementary Table 1). Therefore, it is not possible to directly compare both studies. However, with our experimental procedure, the application of tACS did not enhance low frequency power or the P300 amplitude, which challenges to some extent the robustness of the found effect in the previous study.

One aspect that needs further investigation is to find the optimal P300 time window to be extracted for the online analyses. A limitation of our online analyses was our rather narrowly chosen P300 time frame of 250 to 450 ms, since in four datasets the averaged ERP peaked maximally beyond our chosen P300 time frame. Therefore, for those four participants, the P300 latency, which is used for adjusting the stimulus presentation during the VOT, was not accurate enough. On the other hand, selecting a larger P300 time frame might have led to maximum peaks that fall below (e.g., <200) or exceed (e.g., >600) the usual P300 time window. Hence, future studies might expand the P300 time frame to 250–600 ms targeting ADHD patients.

Another caveat is that our study did not allow for full experimenter blinding, given that the neurostimulator had to be manually adjusted. Hence, an experimenter bias cannot fully be precluded. Therefore, for future studies, it would be helpful to control the neurostimulator automatically instead of manually entering the stimulation parameter.

Another limitation of our study is that our sample size is, unfortunately, not large enough to also allow for ADHD subtype analyses. Such an analysis would have been very interesting, though, because it could be that not all ADHD patients, but at least a certain ADHD subtype or subgroup of ADHD patients (e.g., the predominantly hyperactive/impulsive subtype) benefit from our tACS application. In addition, a sub analysis of patients with certain comorbidities may also have been interesting to look at, since our sample included, for example, ADHD patients with comorbid mild to moderate affective disorders or anxiety disorders. Similarly, the sample we collected may not have been large enough to detect even small tACS-induced changes. In this case, however, the question arises whether these undetected effects are clinically relevant.

Although ERP data give valuable insights into cognitive processing of ADHD patients, it is important to bear in mind that it is still unclear whether the P300 amplitude decrease in ADHD (41–48) is a cause, consequence, or compensatory process. Although first explanation attempts have been put forward (128), further studies are clearly necessary to shed more light on this unresolved question.

Moreover, a question that remains unanswered in our study is the question of possible tACS long-term effects. In particular, our study cannot exclude the possibility that the tACS effects we expected do not occur immediately, but perhaps not until after several sessions. For example, in the study Farokhzadi et al. (86), where alpha-tACS achieved higher reductions in inattention and impulsivity than Ritalin, the effect was measured after 24 sessions. Therefore, it would be interesting to compare various tACS conditions over more than one session. In this respect, it is also conceivable to vary the stimulation frequencies or electrode montages.

In addition, it should be considered that the application of tACS is accompanied by a large artifact in EEG data. It is a major challenge to recover artifact-free brain signals during tACS because it hinders direct insights into electrophysiological processing during stimulation. So far, current computational approaches still fail to obtain artifact-free data (129–132). In the future, however, it would be interesting to analyze EEG data during actual stimulation to lighten the current black box.

## Conclusion

In conclusion, our study cannot provide further evidence that tACS can increase the P300 amplitude in ADHD patients and that by such P300 amplification an immediate improvement of neuropsychological attention parameters can be achieved. However, we found a possible effect of our tACS stimulation on a late ERP component and a positive correlation between this component and the participants’ VOT RTs that both warrant further investigation. Moreover, our chosen setup included many actuation parameters (e.g., stimulation intensity, electrode mounting, waveform type) that could have been set differently. Therefore, there are still many alternative parameter settings for the application of tACS that can be tested and that may potentially yield more promising results.

## Data Availability Statement

The anonymized raw data supporting the conclusions of the article will be made available by the authors, without undue reservation.

## Ethics Statement

The studies involving human participants were reviewed and approved by the Medical Ethics Committee of the University of Bonn. The patients/participants provided their written informed consent to participate in this study.

## Author Contributions

KK and CB designed the experiment under the supervision of NB, AP, and CH. CB conducted the tACS electrode simulations. KK collected and analyzed the data under the supervision of NB and CH, and intervision with CB. KK and NB wrote major parts of the manuscript. BA and HR recruited ADHD patients. AW, BS, CB, AP, SL, HR, BA, and CH contributed to reviewed and edited the manuscript. All authors contributed to the article and approved the submitted version.

## Conflict of Interest

The authors declare that the research was conducted in the absence of any commercial or financial relationships that could be construed as a potential conflict of interest.

## Publisher’s Note

All claims expressed in this article are solely those of the authors and do not necessarily represent those of their affiliated organizations, or those of the publisher, the editors and the reviewers. Any product that may be evaluated in this article, or claim that may be made by its manufacturer, is not guaranteed or endorsed by the publisher.

## Footnotes

1
https://www.drks.de/

2
https://github.com/sccn/labstreaminglayer
