# Supplementary material for: P300 Modulation via Transcranial Alternating Current Stimulation in Adult Attention-Deficit/Hyperactivity Disorder: A Crossover Study
Source: Front Psychiatry. 2022 Jul 18;13:928145. doi: 10.3389/fpsyt.2022.928145 (PMC9339709; doi:10.3389/fpsyt.2022.928145)
Supplement: Supplementary file 1 [file Data_Sheet-1.DOCX]

Supplementary Material

## Supplementary Figures


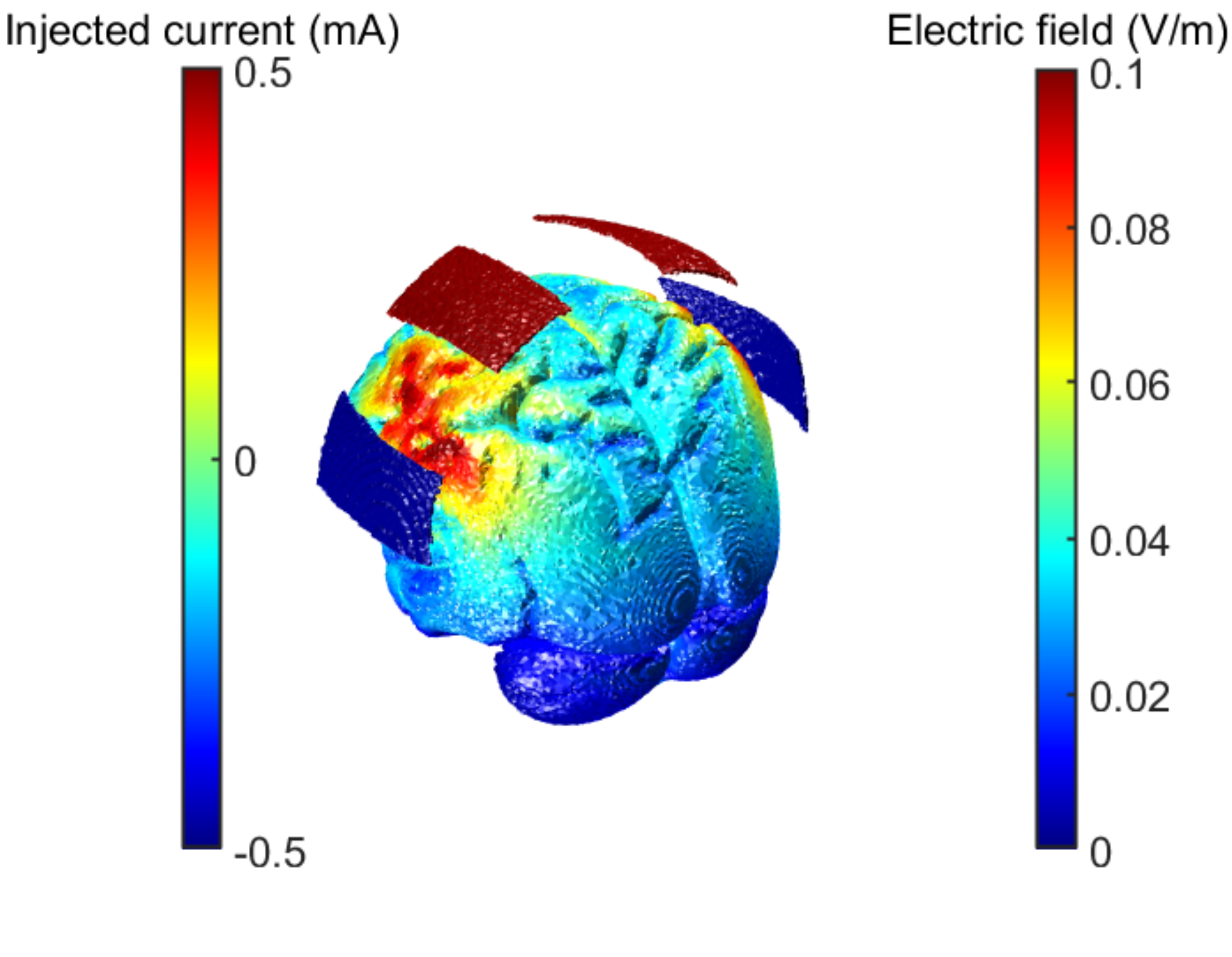


SM Figure 1. Electrode simulations. tACS electrode simulation via ROAST Toolbox.

##
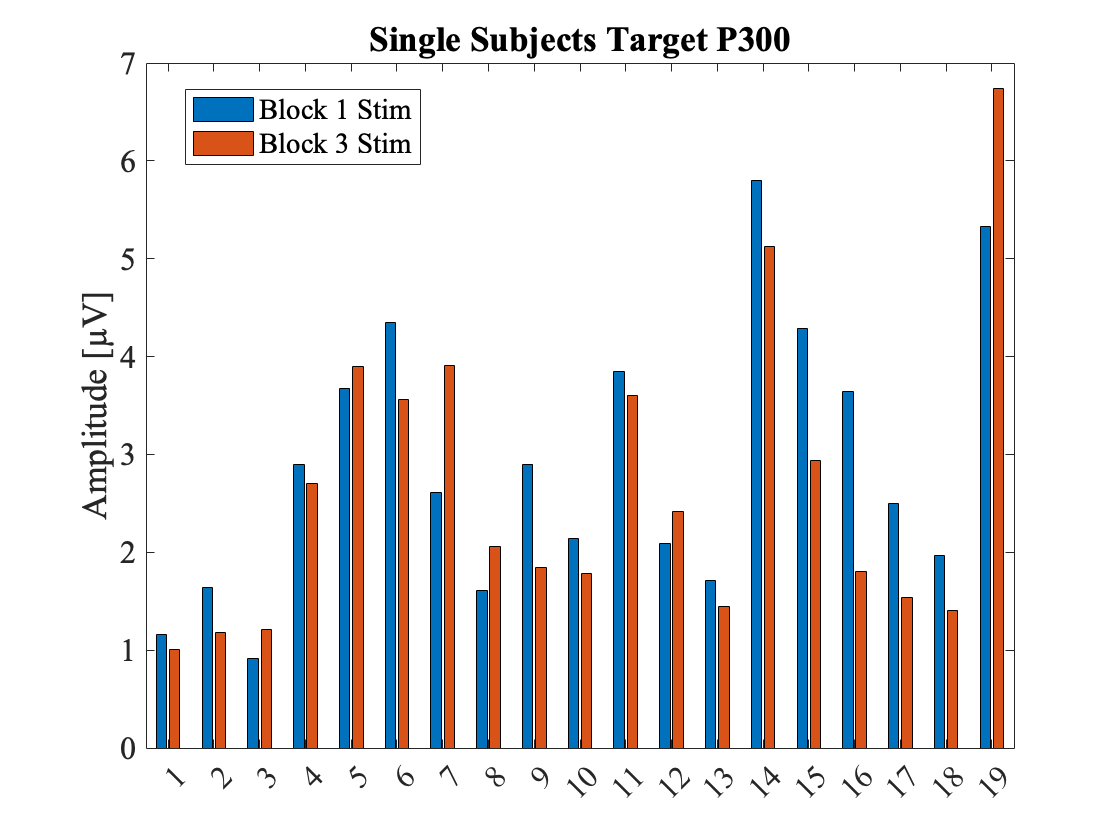


SM Figure 2. Single Subject mean amplitude. Single subject analysis for mean P300 amplitude. On the x-axis, all *n* = 19 participants are shown. Blue bars depict pre-EEG measurements (pre actual stimulation), while orange bars depict the post EEG measurements (post actual stimulation).


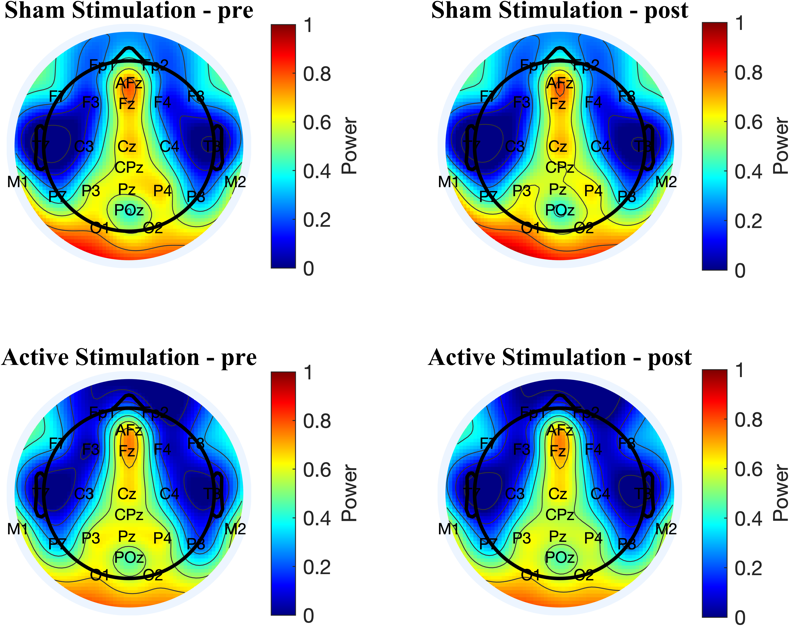


SM Figure 3. Topography plots of the wavelet analysis averaged over the time window 250-550 ms after stimulus onset.

## Supplementary Tables

|  | **Dallmer-Zerbe et al. 2020** | **Kannen et al. 2022** |
| --- | --- | --- |
| ***Study Design*** | Between-Design (*N* = 18) | Crossover Design (*N* = 19^*^) |
| ***Randomization*** | Random group allocation | Counterbalancing |
| ***Visual Oddball Task (VOT)*** | Classical visual oddball task with letters; Stimuli: ‘X’ and ‘O’ | Adjusted visual oddball task with 2° tilted left or right gabor stimuli |
|  | One finger response: Response only to target stimulus ‘X’ | Two finger response: response to every stimulus |
|  | 1000 ms stimulus duration | 500 ms stimulus duration |
| ***Transcranial alternating current stimulation (tACS)*** | Ag/AgCl-ring electrodes | Rubber electrodes |
|  | Twelve ring electrodes ∅ ~ 10 mm | Four electrodes 7 x 3.5 cm |
|  | Electrode positions at C3, C4, CP3, CP4, P3, P4 and T7, T8, TP7, TP8, P7, P8 | Electrode positions above C1/C2 and C5/C6 |
| ***tACS electrode simulation*** | MNI standard brain; Field strength of ~ 0.1 V/m in parietal and temporal cortices | MNI standard brain; Field strength of ~ 0.1 V/m in parietal and temporal cortices |
| ***EEG-System*** | 34 electrodes, BrainAmp amplifier (Brain Products, Gilching, Germany)  1000 Hz sampling rate | 24 electrodes, Smarting® (mBrainTrain®, Belgrade, Serbia)  500 Hz sampling rate |
| ***Online EEG-Analysis*** |  |  |
| *Filtering* | LPF: 20 Hz, HPF: 0.5 Hz | LPF: 40 Hz, HPF: 0.1 Hz |
| *Epoching* | -3 to 4 s around target onset | -2 to 5 s around target onset |
| *Baseline Correction* | -50 to 0 s | -2 to 0 s |
| *P300 time window extraction* | 0-900, 300-600 ms respectively | 250-450 ms |
| *Channel of interest* | Pz | Pz |
| *Artifact identification/removal* | Visual inspection of target trials, rejection of trials including eye-blinks | ICA + Non-stereotypic artefact removal |
| *Frequency analysis* | Wavelet-Transform 1.5-20 Hz, 3 cycles | Power spectrum 1-8 Hz |
|  | Frequency Resolution 0.5 Hz | Frequency Resolution 0.1 Hz |
|  | Time resolution: 0.024 s | Time resolution: 0.124 ms |
|  | Event-related spectral perturbation (ERSP) within ± 150 ms time window around individual P300 peak latency | Maximum power spectrum between 1-8 Hz within ± 200 ms time window around individual P300 peak latency |
| ***EEG-Preprocessing*** |  |  |
|  | n.a. | Merging pre- and post- intervention blocks |
| Re-referencing | Common average | Common average |
| *Down-Sampling* | 500 Hz | 250 Hz |
| *Filtering* | 8 Hz LPF | 0.5-40 Hz, detrending |
| *Artifact identification/removal* | n.a. | Noisy channel removal and spherical interpolation |
|  | ICA | Epoching (2 s), ICA (‘extended version’) |
| ***Offline EEG-Analyses*** |  |  |
| ***P300-Analysis*** |  |  |
| *Filtering* | 8 Hz LPF | 6 Hz LPF |
| *Epoching* | − 3 to 4 s around target onset | -0.5 to 1.5 s around target onset |
| *Baseline-Correction* | − 50 to 0 ms | -0.5 to 0 s |
| *Artifact removal* | n.a. | Reject channels > 150 μV in > 15 % epochs; Epoch > 10 bad channels were excluded; spherical interpolation |
|  | Maximum P300 peak between 300-600 ms | Mean P300 amplitude (200-550 ms time frame) |
| ***Frequency-Analysis*** | Event-related spectral perturbation (ERSP) | Continuous wavelet transformation (CWT) |
| *Frequency Resolution*  *Time Resolution* | 0.25 Hz  0.014 s | 0.25 Hz to 6 Hz in 47 steps on a log scale  0.004 ms |
|  | Within ± 150 ms time window around individual P300 peak latency | Mean power spectrum and maximum power between 250-550 ms |
| ***Outcome Variables*** | Mean Reaction Time  Reaction Time Variability  Omission Errors  Relative change P300 amplitude  P300 latency  Maximum delta/theta power | VOT Mean Reaction Time  VOT Reaction Time Variability  VOT Omission error rate & Commission error rate  D2 Processing speed, omission & commission errors, concentration performance  P300 mean amplitude  Mean delta/theta power |
| ***Statistics*** | Mann–Whitney test | ANOVA |

SM Table 1. Comparison between the previous study by Dallmer-Zerbe et al. (2019) and our study. Abbreviations: n.a. = not applicable, LPF = Low pass filter, HPF = High pass filter, ICA = independent component analyses, VOT = visual oddball task. ^*^Out of 20 participants who completed the entire experiment, one participant had to be excluded from the analyses due to incorrect task execution. Hence, 19 participants remained for analyses.

| **d2 Task** | **Predictor** | ***df*** | ***F*** | ***p*** | ***η_p_^2^*** |
| --- | --- | --- | --- | --- | --- |
| **Processing Speed** |  |  |  |  |  |
|  | Block | 1,15 | 40.32 | < .001 | .73 |
|  | Intervention | 1,15 | 0.041 | .842 | .00 |
|  | Block*Condition | 1,15 | 0.09 | .772 | .01 |
| **Concentration Performance** |  |  |  |  |  |
|  | Block | 1,15 | 23.80 | < .001 | .61 |
|  | Intervention | 1,15 | 0.00 | .998 | .00 |
|  | Block* Intervention | 1,15 | 0.68 | .443 | .04 |
| **Omission Errors** |  |  |  |  |  |
|  | Block | 1,16 | 7.78 | .013 | .33 |
|  | Intervention | 1,16 | 0.19 | .672 | .01 |
|  | Block*Intervention | 1,16 | 2.02 | .174 | .11 |
| **Commission Errors** |  |  |  |  |  |
|  | Block | 1,16 | 5.21 | .037 | .25 |
|  | Intervention | 1,16 | 0.02 | .884 | .00 |
|  | Block*Intervention | 1,16 | 1.56 | .229 | .09 |

SM Table 2. ANOVA table d2 attention test.

| **Visual Oddball Task** | **Predictor** | ***df*** | ***F*** | ***p*** | ***η_p_^2^*** |
| --- | --- | --- | --- | --- | --- |
| **Omission Error Rate** |  |  |  |  |  |
|  | Block | 1,18 | 20.13 | <.001 | .53 |
|  | Intervention | 1,18 | 0.08 | .781 | .00 |
|  | Block*Condition | 1,18 | 0.16 | .693 | .01 |
| **Comission Error Rate** |  |  |  |  |  |
|  | Block | 1,18 | 1.61 | .220 | .08 |
|  | Intervention | 1,18 | 0.37 | .548 | .02 |
|  | Block* Intervention | 1,18 | 0.58 | .458 | .03 |
| **d Prime** |  |  |  |  |  |
|  | Block | 1,18 | 17.85 | <.001 | .50 |
|  | Intervention | 1,18 | 0.47 | .501 | .03 |
|  | Block*Intervention | 1,18 | 0.32 | .576 | .02 |
| **Reaction Time** |  |  |  |  |  |
|  | Block | 1,18 | 2.26 | .150 | .11 |
|  | Intervention | 1,18 | 0.14 | .710 | .01 |
|  | Block*Intervention | 1,18 | 2.12 | .163 | .11 |
| **Reaction Time Variability** |  |  |  |  |  |
|  | Block | 1,18 | 2.08 | .167 | .10 |
|  | Intervention | 1,18 | 0.23 | .635 | .01 |
|  | Block*Intervention | 1,18 | 0.65 | .432 | .03 |

SM Table 3. ANOVA table Visual Oddball Task.

|  | **Predictor** | ***df*** | ***F*** | ***p*** | ***η_p_^2^*** |
| --- | --- | --- | --- | --- | --- |
| Maximum P300 Peak |  |  |  |  |  |
|  | Block | 1,18 | 0.40 | .533 | .02 |
|  | Intervention | 1,18 | 0.14 | .715 | .01 |
|  | Block*Intervention | 1,18 | 0.05 | .825 | .01 |

SM Table 4. ANOVA table maximum P300 amplitude.
